# Simultaneous evaluation of anti-EGFR-induced tumour and adverse skin effects in a microfluidic human 3D co-culture model

**DOI:** 10.1038/s41598-018-33462-3

**Published:** 2018-10-09

**Authors:** Juliane Hübner, Marian Raschke, Isabel Rütschle, Sarah Gräßle, Tobias Hasenberg, Kerstin Schirrmann, Alexandra Lorenz, Susanne Schnurre, Roland Lauster, Ilka Maschmeyer, Thomas Steger-Hartmann, Uwe Marx

**Affiliations:** 1 Technische Universität Berlin, Institute of Biotechnology, Department Medical Biotechnology, Gustav-Meyer-Allee 25, 13355 Berlin, Germany; 2 TissUse GmbH, Oudenarder Str. 16, 13347 Berlin, Germany; 3 Bayer AG, Investigational Toxicology, 13353 Berlin, Germany; 4 The University of Manchester, Manchester Centre for Nonlinear Dynamics, Oxford Rd, Manchester, M13 9PL UK

## Abstract

Antibody therapies targeting the epithelial growth factor receptor (EGFR) are being increasingly applied in cancer therapy. However, increased tumour containment correlates proportionally with the severity of well-known adverse events in skin. The prediction of the latter is not currently possible in conventional *in vitro* systems and limited in existing laboratory animal models. Here we established a repeated dose “safficacy” test assay for the simultaneous generation of safety and efficacy data. Therefore, a commercially available multi-organ chip platform connecting two organ culture compartments was adapted for the microfluidic co-culture of human H292 lung cancer microtissues and human full-thickness skin equivalents. Repeated dose treatment of the anti-EGFR-antibody cetuximab showed an increased pro-apoptotic related gene expression in the tumour microtissues. Simultaneously, proliferative keratinocytes in the basal layer of the skin microtissues were eliminated, demonstrating crucial inhibitory effects on the physiological skin cell turnover. Furthermore, antibody exposure modulated the release of CXCL8 and CXCL10, reflecting the pattern changes seen in antibody-treated patients. The combination of a metastatic tumour environment with a miniaturized healthy organotypic human skin equivalent make this “safficacy” assay an ideal tool for evaluation of the therapeutic index of EGFR inhibitors and other promising oncology candidates.

## Introduction

The human epidermal growth factor receptor (EGFR), a member of the erbB family of receptors, is a transmembrane receptor tyrosine kinase expressed in epithelial, mesenchymal and neuronal tissues. It is a key regulator of organ homeostasis mediating physiological cell turnover – proliferation and differentiation – in adults^1^. This receptor is frequently overexpressed in many tumours, such as carcinomas and glioblastoma^2,3^, and serves as a prime target for tumour therapies. Inhibitors of EGFR activation, using monoclonal antibodies or small-molecule inhibitors, have been established successfully and are increasingly being used in first or later line cancer therapy^4^. However, its inhibition induces adverse responses primarily in skin and intestine – the organs with the highest physiological cell turnover. Receptor-mediated (EGFR) adverse cutaneous effects result in dose reduction or discontinuation of treatment, limiting the effectiveness of tumour therapy^5^.

The mechanisms underlying the correlation between the intensity of the adverse effects in skin and the efficacy of tumour treatment are still poorly understood due to the lack of suitable assay formats. Human skin biology differs substantially from that of most laboratory animal species, which hampers a simultaneous evaluation of anti-tumour efficacy and adverse cutaneous effects by *in vivo* animal studies. Standard *in vitro* assays are limited to the evaluation of the anti-EGFR response in either human tumour models or skin equivalents, each in a separate conventional static tissue culture. This restricts cross-talk and perfusion-based pharmacokinetic studies with relevant dosing background for further quantitative *in vitro* to *in vivo* extrapolation. Consequently, preclinical development of novel effective EGFR-inhibitors would require a reliable holistic human assay platform generating both target-mediated efficacy and safety data based on the homeostasis of a patient’s skin and tumour tissue co-culture.

Co-cultures of human microtissues in microphysiological systems (MPS; e.g. body-on-a-chip) emulating organ cross-talk and mechanisms of disease progression are used increasingly for detailed temporal studies of pharmacological effects of drugs^6,7^. A first drug-testing assay based on the co-culture of a rat lung type II epithelial cell line together with either H4IIE rat hepatocytes or the human HepG2/C3A hepatocyte cell line was first described in 2004^8^. Since then, the number of cell types used in such MPS-based suspension or monolayer co-cultures has increased immensely^9^. Furthermore, the degree of complexity of the organ equivalents used in MPS platforms has improved from suspension and monolayer culture to three-dimensional (3D) tissue culture, emulating their human counterparts more precisely. The first successful examples were single organ equivalents for lung alveoli^10^ and various skin equivalents^11^. The latter have been improved into a robust commercially available multi-organ chip (MOC) platform, where human skin biopsies can be co-cultured homeostatically over four weeks with a human 3D spheroid liver model^12^. Subsequently, microfluidic channels of the MOC co-cultures can be endothelialized^13^. A roadmap towards full integration of MPS into industrial use with regulatory acceptance within the next few decades has been outlined recently by representatives of leading academic groups, MPS technology providers, large industries and regulatory bodies from major countries^14^. Intensive industrial adoption of such MPS-based assays fitting the different purposes within the drug development cycle is ongoing^15^.

We established a novel human tumour–skin co-culture assay to evaluate anti-EGFR antibody effects on both tumour and human skin tissue, the latter being the site of target-mediated adverse effects in patients. We investigated the impact of repeated cetuximab (trade name: Erbitux®) exposure on the systemic behaviour of the co-culture and individual tissue responses. We present here an MPS-based co-culture assay that has the potential to provide a platform for evaluation of the therapeutic window of drug candidates.

## Results and Discussion

Lung cancer is the most common cause of cancer death in the United States^16^. Activation of EGFR-tyrosine kinases is a key for lung cancer progression^17^. The EGFR-targeting therapies, such as monoclonal antibodies, specific tyrosine kinase inhibitors or a combination of both, are a promising approach in the pipeline^4,18^. Innate or acquired resistance of distant metastatic tumour foci to EGFR-targeted therapies after primary tumour resection and severe adverse effects in skin are major challenges for therapeutic success^17,19^. Preclinical *in vitro* assays for the evaluation of EGFR-targeting lung cancer therapies lack the ability to investigate efficacy simultaneously on distant tumour metastasis and adverse skin reaction at their interplay. Prime requirements for such an assay tool are stable long-term homeostasis of a human metastatic lung tumour model and a human skin equivalent, and low fluid flow forces in and microscopic access to the tumour microtissue compartment.

### Hybrid Multi-Organ-Chip (HMOC) design and microfluidic set up

**Figure 1:**
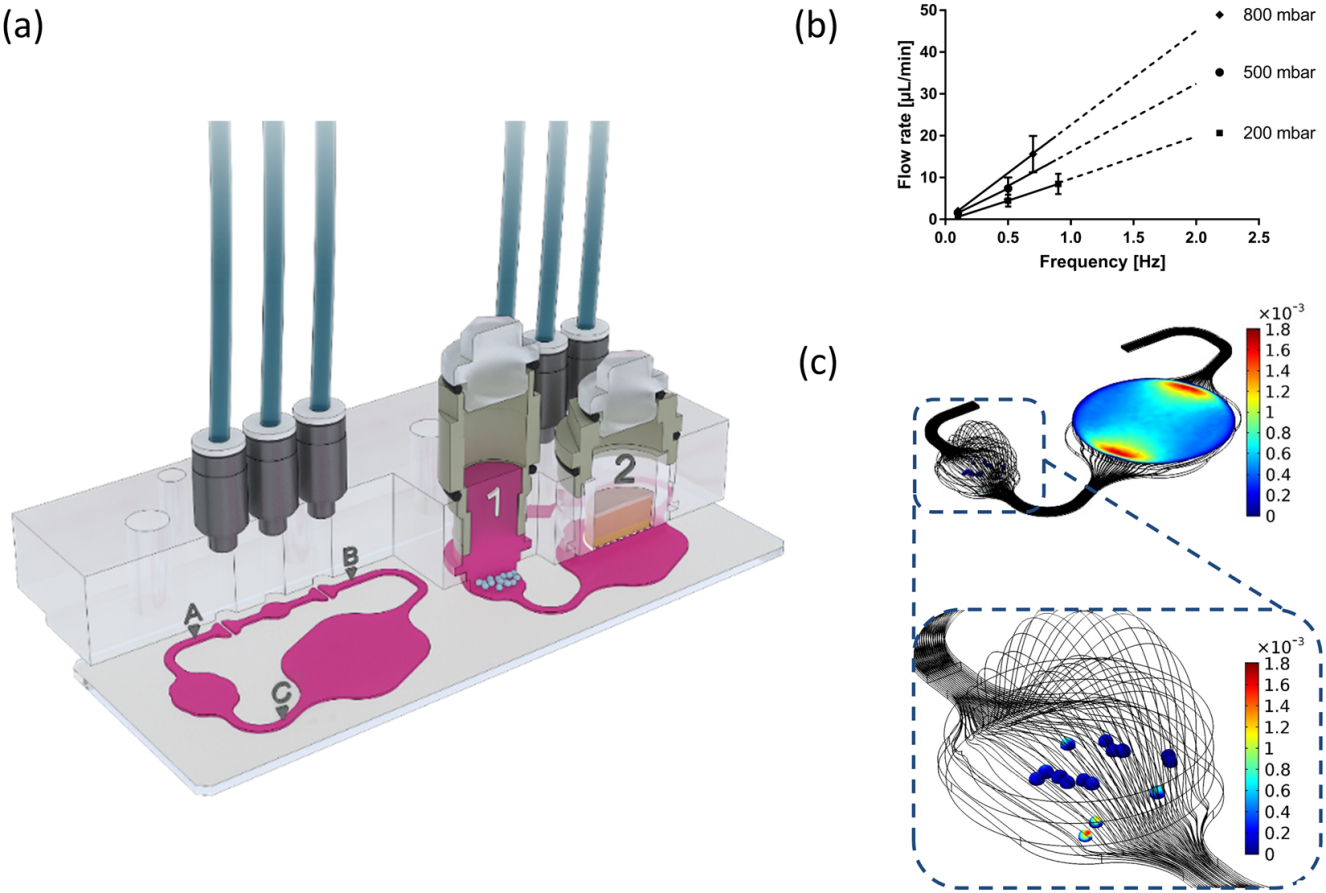
The microfluidic device at a glance. (**a**) Cross-sections of the HMOC. The right cross-section illustrates the tumour (1) and skin (2) microtissue compartments schematically. The left cross-section shows the interrelationship between the compartments and the microparticle imaging points (A–C). The peristaltic three-valve micropump lies between A and B. (**b**) Relationship between frequency and average flow rate at three different pressures (n = 10 for each data point). A linear factorial model was generated from the data acquired and extrapolated to 2 Hz (dashed lines). (**c**) Wall shear stress at the surface of the tumour microtissues and at the base of the skin Millicell® standing insert (colours in dyn/cm^2^) and streamlines (black lines) in the chip.

We designed a new microphysiological HMOC accommodating two identical microfluidic circuits, each interconnecting a 96- with a 24-well sized culture compartment. Henceforth, any tissue model cultured on standard 24-well based cell culture inserts can be easily transferred and cultured for the desired culture time in this new MOC platform. In this study, a co-cultivation of tumour and skin microtissue was established. Figure 1a illustrates the system at a glance. Microscopic access to each and every area of the HMOC allows the tracking of the metastatic outgrowth of the tumour microtissues through daily imaging and facilitates in-depth fluid flow analyses at spots A, B and C on the chip. Recording the fluid flow at three points was necessary to acquire valid velocity values and to determine irregularities. The skin culture compartment hosts a commercially available standard 24-well Millicell® standing insert-based full-thickness human skin microtissue with a surface of 0.6 cm^2^ at the air–liquid interface. This corresponds to 1/30,000 of the average skin surface of a human adult. However, the skin could also be replaced by any other organ equivalent, produced in, for example, 24-well Millicell® standing inserts, such as intestine or lung equivalents, depending on the toxicity target to be analysed. The tumour microtissue compartment is designed to mount a minimum of 15 homogeneous 3D tumour spheroids and support their adherence-induced metastatic outgrowth within the compartment over five days of co-culture. The HMOC has been designed to circulate 150–180 μl fluid in the channel system of each circuit to match a surrogate blood volume, which is 30,000-fold smaller than the original blood volume of an adult human. Additionally, the HMOC consists of an artificial media reservoir of 600 μl above the tumour microtissue culture compartment. Such a reservoir obviously does not exist *in vivo*. However, in the HMOC, it is necessary to provide sufficient nutrition to the tissues for daily feeding.

A peristaltic micropump generates a clockwise pulsatile flow at a wide range of pumping frequencies, generating an almost linearly correlating mean fluid flow velocity (Fig. 1b). The pumping frequency of 0.5 Hz used to operate the HMOC for the assay corresponds to 30 “heart-beats” per min (approximately 43% of the physiological value of an adult at rest). At this frequency, the medium flow rate corresponds to 8 µl/min (Fig. 1b), supporting a complete medium turnover in each HMOC circuit every 2 h. The haemodynamic forces in the bloodstream in humans reach 0.5–4.0 dyn/cm^2^ in the venous circulation and 4.0–30.0 dyn/cm^2^ in the arterial circulation^20^. The wall shear stress in the microfluidic channels reaches up to 2.7 dyn/cm² and, therefore, lies in the physiological range of the venous circulation. Former studies identified mechanical stress as an important regulator of apoptosis and autophagy in circulating tumour cells^21–23^. By comparison, a tumour cell in metastatic growth after extravasation from the circulation would experience much lower forces. Therefore, the system was adjusted to low shear stresses in the tumour compartment. The mean wall shear stress at the surface of the tumour spheroids is of the order of 10^−4^ dyn/cm^2^ (Fig. 1c; blue colour). If the microtissues are close to the inlet or outlet of the compartment, the shear stress is slightly higher (Fig. 1c; yellow and red colour). Overall, the applied culture conditions enable an adequate distribution of nutrients to the microtissues and, simultaneously, shield the tumour microtissue from human bloodstream-like shear stress.

### Preformed skin and tumour microtissues

**Figure 2:**
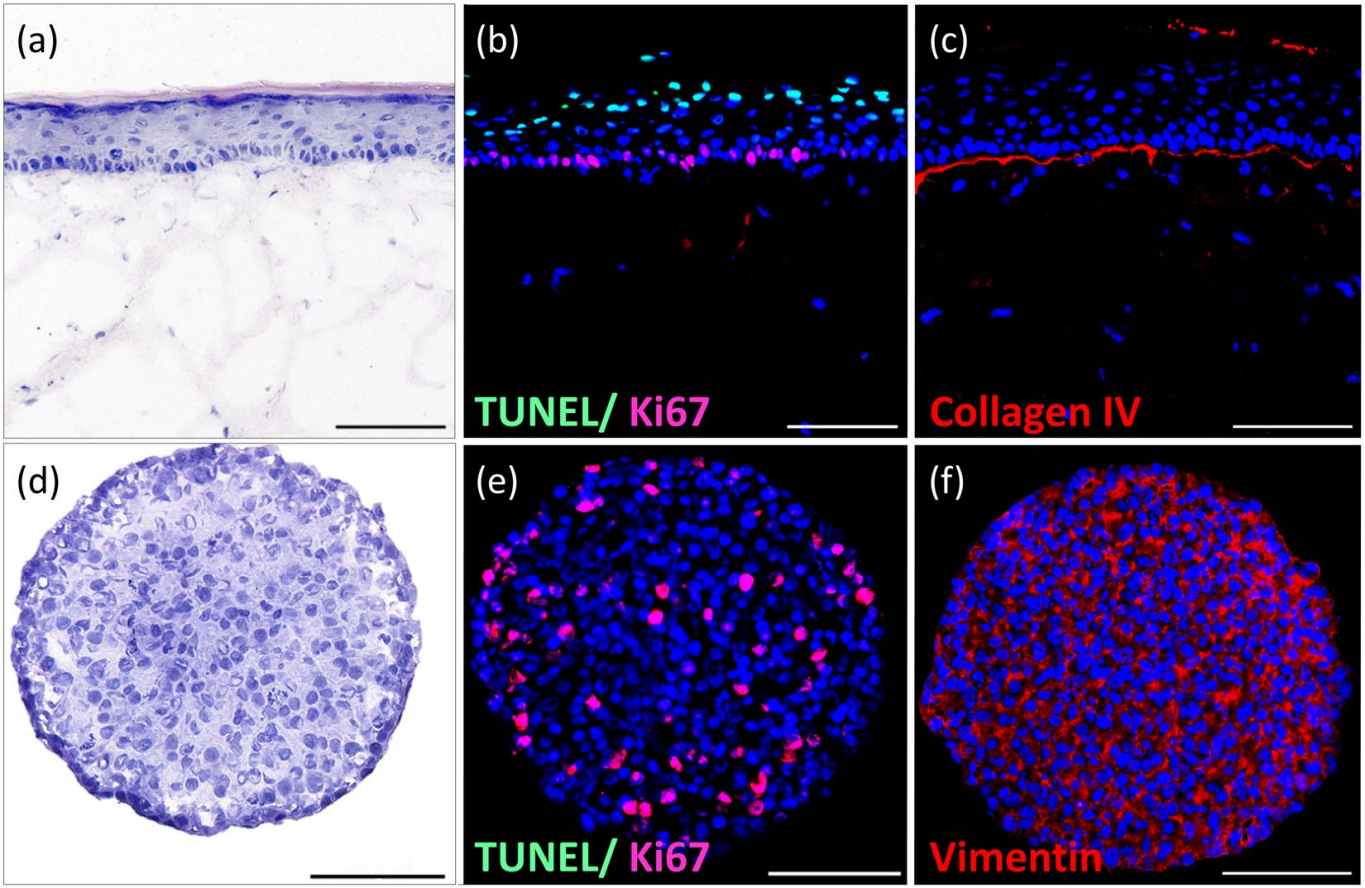
Morphology of *in vitro* generated skin microtissue (**a**–**c**) and tumour microtissue (**d**–**f**). (**a**) Haematoxylin and eosin (H&E) staining of the physiological architecture of a human full-thickness skin microtissue on day 16 of air–liquid culture under static conditions showing the columnar basal keratinocyte layer, a stratified stratum corneum and the layers in-between. (**b**) Immunofluorescent TUNEL (green) and Ki67 (red) staining indicating proliferative keratinocyte stem cells in the basal layer (red) and the apoptotic (cornifying) cells (green) on the upper skin layer. (**c**) Collagen IV staining of the basement membrane of the skin. (**d**) H&E staining of homogeneously distributed identical H292 cells throughout the tumour spheroid. (**e**) Immunofluorescent TUNEL (green) and Ki67 (red) staining indicating healthy tumour microtissue status containing a significant fraction of growing tumour cells (red). (**f**) Vimentin (red) staining of the tumour microtissue showing fibroblastic properties. (**b**,**c**,**e**,**f**) Nuclei stained with DAPI (blue). Scale bar: 100 µm.

A standardized commercially available human full-thickness skin equivalent composed of a dermal part containing matrix-grown human fibroblasts and an epidermal part built from human keratinocyte layers fabricated in standard 24-well Millicell® standing inserts was used for the HMOC assays. The keratinocyte growth factor and epidermal growth factor were routinely supplemented at a well-optimised ratio to the culture to sustain human full-thickness skin equivalents^24^. A stratified stratum corneum, continuously generated by cornifying keratinocytes, constitutes the air–liquid interface (Fig. 2a). The columnar basal keratinocyte layer at the interface to the dermal part comprises regularly distributed keratinocyte stem cells, which are stained positively for Ki67 expression (Fig. 2b, red staining), continuously renewing the upper keratinocyte layers. Additionally, a few TUNEL positive keratinocytes could be detected at the upper epidermal layer (Fig. 2b, green staining). They indicate a physiological keratinization within the epidermal differentiation to produce the stratum corneum where cell death of keratinocytes is mandatory^25^. Furthermore, the presence of a basement membrane, shown by positive Collagen IV staining (Fig. 2c), represents epidermal integrity. Such full-thickness skin equivalents reflect the basic architecture and turnover of the human epidermis and are widely used for testing purposes^26^. It is well-known that the epidermal growth factor impacts growth and differentiation of the epidermal layer via EGFR in such skin equivalents, eventually mimicking wound-healing processes^24^. Therefore, we anticipated such skin equivalents to be reflective of potential anti-EGFR antibody effects in human skin.

We selected the human NCI-H292 cell line for the establishment of the tumour microtissue in the HMOC assay because of five essential features: its epithelial lung tissue origin, metastatic track record, capability to undergo epithelial to mesenchymal transformation, compliance with existing 3D spheroid formation protocol and availability of a standardized monolayer efficacy test for anti-EGFR antibodies. The cell line has been derived from a cervical lymph node metastasis of a high-grade pulmonary mucoepidermoid carcinoma from a 32-year-old Negro female^27–29^. H292 cells, therefore, have been naturally selected for aggressive malignant properties. Generally, only 0.01% of circulating tumour cells have a proliferation potential to form macrometastasis^30^. They expose aggressive malignant properties and form the distant metastatic colonies^31,32^. H292 cells retain epithelial mucoepidermoid characteristics and, simultaneously, express fibroblastoid properties. This is typical for epithelial to mesenchymal transformation, a common characteristic for pleomorphic carcinomas of the lung^33^. Finally, robust protocols to generate 3D H292 cell spheroids for *in vitro* evaluation have been established^34,35^. Using an ultra-low attachment plate culture protocol, homogeneous spheroids of a median diameter of ~345 µm composed of approximately 10,000 cells per spheroid could be formed within three days. At this spheroid size, the cells are evenly distributed throughout the entire spheroid (Fig. 2d). No apoptotic core (shown by negative TUNEL staining) could be observed, indicating an efficient supply of nutrients and oxygen across each spheroid. Furthermore, a subfraction of ~27% Ki67-positively stained cells is evenly distributed inside the spheroid, indicating a certain growth capability (Fig. 2e). In addition, positive vimentin staining specified fibroblastic properties of lung tumour spheroids (Fig. 2f). Fifteen such spheroids are seeded into the tumour culture compartment, each mimicking an individual disseminated metastatic tumour focus with a cumulative surface area of 5.6 mm^2^ on a total attachment surface of the compartment of 33.2 mm^2^. The single cells have a median diameter of ~ 22 µm per cell and a histological appearance comparable with epidermoid type of cells in non-small cell lung cancer (NSCLC)^36^.

### HMOC-based anti-EGFR antibody assay

A robust five-day skin-tumour co-culture regimen has been established and qualified by five series of experiments involving 69 HMOC co-culture circuits. The assay longevity was limited to five days for two reasons: 1) Robust 3D H292 cell spheroid cultures have been reported in static ultra-low attachment plate culture for that time frame^35^; and 2) cetuximab half-life time is around five days.

**Figure 3:**
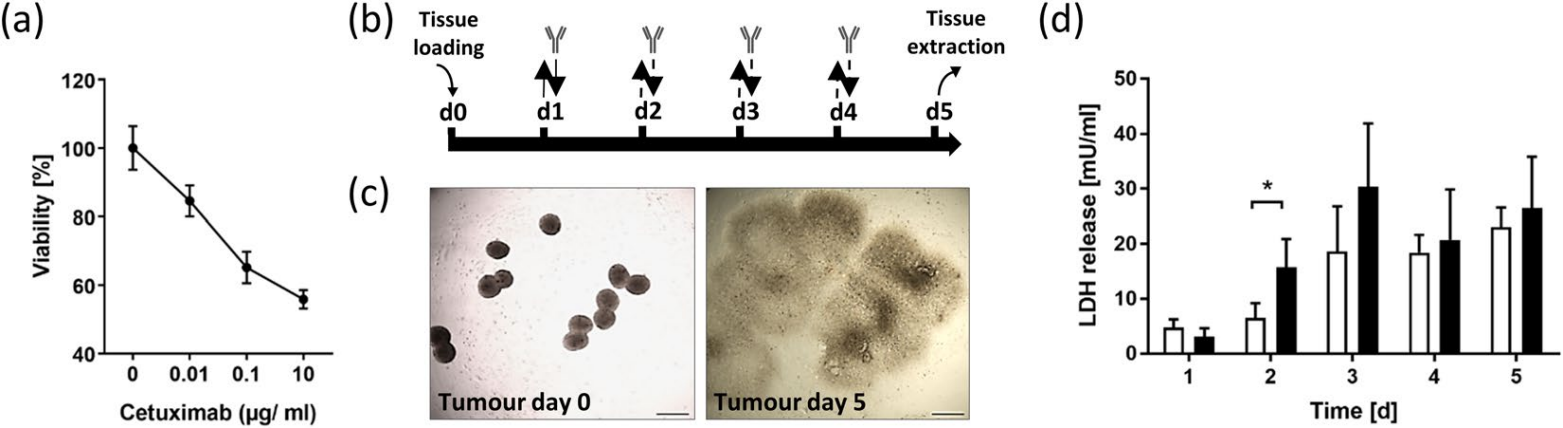
Static monolayer tumour proliferation assay (**a**) and tumour behaviour in five-day chip co-culture (**b–d**). (**a**) Dose-dependent cytotoxicity of cetuximab on H292 cells in static monolayer culture, n = 6. (**b**) Co-culture regimen illustrating total media volume exchange (), half media volume exchange () and antibody exposure time points (). (**c**) Representative tumour culture compartment section illustrating a typical arrangement of tumour spheroids at day 0 and 3D fried egg shape after five days. Scale bar: 500 µm. (**d**) LDH release in untreated control group (white bars) and in antibody exposure group (black bars) over five days of chip culture, n = 4. *p < 0.05 using unpaired two-tailed Student’s t test. Data shown as mean + SD.

Prior to starting the HMOC assay, a validated standardized 2D efficacy assay for H292 cell treatment with cetuximab was performed to demonstrate the H292 cell line sensitivity to the drug for the cell batch used (Fig. 3a). Our data are in line with values reported from the literature, demonstrating a dose-dependent cell death of up to 56% at the highest dose. Exposure to higher doses of cetuximab could not further increase this percentage of apoptotic H292 cells, although more than 90% of the cells in monolayer express EGFR^37,38^. Thus, the monolayer assay proved the pleomorphic nature of the H292 cells generating distinct subpopulations of cells under well-defined culture conditions. Furthermore, it proved the suitability of the H292 cell culture lot used for the experiments.

We were able to select a cell culture medium universally supporting both skin and tumour microtissue maintenance over the entire culture period at a daily feeding regimen, illustrated in Fig. 3b. The co-cultures have been divided into two groups: I) the control group, where no antibody was applied during the daily medium exchange; and II) the antibody exposure group, where cetuximab was applied repeatedly over 4 days. Cetuximab is a chimeric monoclonal IgG1 antibody (molecular weight of 145.781.6 g/mol; biological half-life of 114 h) specifically binding EGFR with an affinity 5 to 10 times higher than that of endogenous ligands, thereby, blocking their binding^39^. It has been shown to bind to NSCLC and colorectal cancer from patients, and to H292 tumour cells^38^. It also triggers antibody-dependent cell-mediated cytotoxicity *in vivo*^40^. The antibody has been authorized for the treatment of a number of cancer indications since 2003. Exposure to cetuximab antibody was customized from a treatment cycle of cancer patients to fit the HMOC assay. Doses have been extrapolated from human exposure regimens established for the use of cetuximab in head and neck cancer^41^. At day 1, these patients receive a single intravenous dose of 400 mg/m^2^ of skin surface. Considering that the skin surface of each circuit in the HMOC has a surface of 0.6 cm^2^, this would correspond to a systemic application of 24 µg cetuximab per circuit. Patients then, from day 7 onwards, receive radiotherapy accompanied by weekly infusions of 250 mg/m^2^ cetuximab, corresponding to 15 µg per circuit of an HMOC. However, due to the lack of the other organ equivalents, the total biomass in the HMOC is lower than *in vivo*. Therefore, we have reduced the first exposure to 10 µg at day one. The further application regimen has been set to a daily treatment of 5 µg per circuit combined with a 50% medium exchange (Fig. 3b), which counts for roughly a third of the human exposure. Absorption of cetuximab into the PDMS layer of the HMOC was investigated by performing a non-specific binding test using a cell-free HMOC. The analysis revealed a stable antibody concentration certifying no antibody reduction due to absorption (data not shown).

H292 cell spheroids immediately attached at their discrete points of settlement (Fig. 3c). Under mild convective medium flow (shear stress) in their culture compartment, each spheroid formed a distinct metastatic focus, collectively occupying nearly the entire compartment surface at day five of the HMOC co-culture. The distinctive fried egg shape of the H292 spheroid-generated metastatic tumour foci, seen in all tumour compartments, points to the solid tumour mass (“egg yolk”) of the metastatic foci surrounded by a flattened tumour microtissue (“egg white”) invading any free surface (Fig. 3c).

The distribution of antibodies in patient’s tumour metastases depends on tumoural pathophysiology and antibody characteristics, such as affinity and molecular weight^42^. Animal studies have shown that most of the therapeutic antibodies were concentrated in a region within 40 µm (or two tumour cell diameters) of the tumour vessels^43^. We calculated antibody residence times by integrating 1/v (v: magnitude of velocity) over streamlines in a contact zone of 50 µm around the tumour microtissues, which corresponds to about two diameters of the H292 cells. This contact zone distance is much bigger than the molecule size of an antibody (~10 nm) but provides the first rough insight into antibody–tumour interaction potential at reasonable computational modelling costs. This model revealed an increase of the median residence time of the antibody molecules in proximity to the dispersed metastatic tumour microtissues from 4.5 min at day 0 to 8 min on day 3 and 11 min on day 5. This increase of the residence time is due to the rapidly changing architecture of the metastases, which then provides an increased exposure surface to the convective fluid flow on day 5 compared to day 0 and mimics the situation in patients when metastases are extending rapidly in size.

The LDH served as a marker for cell death in the co-culture. Antibody-treated co-cultures showed a significantly higher systemic release of LDH 24 h after the first cetuximab application, indicating a toxic antibody-dependent effect (Fig. 3d). Afterwards, LDH values stabilized to a similar range of 20–30 mU/ml within the control group and the antibody exposure group indicating a stable turnover. These values generally count for a high viability of the microtissues, which can generate LDH values of ~2200 mU/ml at 100% of cell death (data not shown).

### Effects of anti-EGFR antibody treatment on tumour microtissues in co-culture

**Figure 4:**
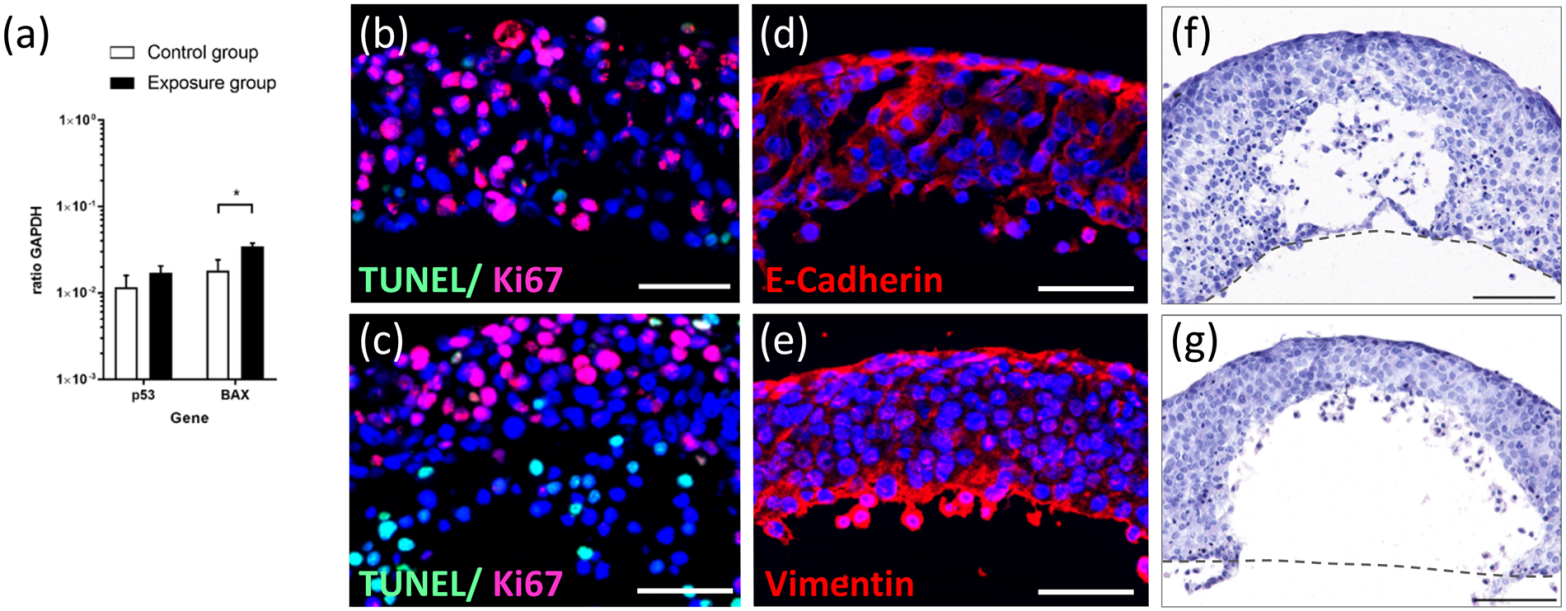
Effects of cetuximab treatment on tumour microtissue at day 5 of co-culture. (**a**) Gene expression of tumour protein p53 and apoptosis regulator BAX in untreated control group (white bars, n = 3) and in antibody exposure group (black bars, n = 2–3). *p < 0.05 using unpaired two-tailed Student’s t test. Data shown as mean + SD. (**b**) Immunofluorescent TUNEL (green) and Ki67 (red) staining of untreated control group indicating especially proliferative cells throughout the tumour microtissue. (**c**) Immunofluorescent TUNEL (green) and Ki67 (red) staining of antibody exposed group demonstrating the occurrence of proliferative and apoptotic cells. (**d**) Immunofluorescent staining of E-cadherin (red). (**e**) Immunofluorescent staining of vimentin (red). E-cadherin and vimentin staining are shown exemplarily only for exposed tumour microtissues. Nuclei stained with DAPI (blue). Scale bar: 50 µm. (**f**) H&E staining of untreated control group indicating two types of tumour cell nuclei. (**g**) H&E staining of antibody-exposed group showing similar nuclei differences. Dashed line (grey) indicates chip culture surface during co-culture. Scale bar: 100 µm.

Cetuximab binds to the extracellular domain of EGFR, which induces receptor internalization, degradation of the receptor leading to the downregulation of EGFR at the cell surface. Therefore, autophosphorylation and activation of the receptor is prevented and further downstream signal transduction pathways are inhibited^44^. This can lead to proliferation inhibition and apoptosis induction in tumour cells^45^. We, therefore, checked for genes related to apoptosis induction. The tumour suppressor gene p53 plays a crucial role in tumour initiation and progression. As a transcription factor, it can regulate downstream target genes such as BAX, a pro-apoptotic protein, belonging to the Bcl-2 family. Gene expression analysis of tumour microtissues revealed an increase of p53 and a significant upregulation of BAX within the antibody exposure group in comparison to the control group (Fig. 4a). This data indicates that the tumour microtissue responds to cetuximab exposure with an altered gene profile towards pro-apoptotic signalling. Nevertheless, immunohistochemical analysis of tumour microtissues showed a considerable Ki67 expression on the outer tumour edges in both culture conditions (at a depth of 50–100 µm), indicating a certain proliferation potential (Fig. 4b,c). These findings support the morphological observation that H292 cells of the control group and the antibody exposure group migrated expansively during the five-day co-culture independent of the treatment applied. However, in comparison to the control group, cells of cetuximab-exposed tumour microtissues showed additional apoptotic cells positively stained for TUNEL (Fig. 4c) within the antibody exposure group. Furthermore, tumour microtissues of the control group and the antibody exposure group showed distinct expression of E-cadherin at the edge connected to the convective fluid flow (Fig. 4d). Together with the vimentin expression (Fig. 4e) at the same edge, two markers for mucoepidermoid and fibroblastoid properties are present within our pleomorphic H292 cell-based tumour microtissues. This might be a result of epithelial to mesenchymal transition, well described for lung carcinomas^33^. We assume that our tumour microtissues have, therefore, the potential to create a constant high interstitial fluid pressure inside, well described in literature for aggressive malignant tumour metastasis^46,47^. The consistent and reproducible formation of areas free of tumour cells in the central part of the tumour microtissues emulates tumour necrosis areas in NSCLC malignancy areas^33^. In addition, analysis of H&E staining revealed differences in tumour cell nuclei appearance (Fig. 4f,g). There are two potential explanations for the evident survival of proliferating tumour cells under cetuximab treatment in comparison to the monolayer proliferation assay: 1) The high interstitial pressure inside the tumour hinders antibody penetration from the convective flow into the tumour^46^, and 2) decreased EGFR receptor expression and density together with a higher intra-tumoural basal EGFR phosphorylation decreases antibody sensitivity^35^. It remains unclear whether the dead proportion of tumour cells in the control and antibody exposure group are related to antibody-specific death or to intra-tumoural death due to oxygen limitation.

Based on these factors, our tumour spheroid-based microfluidic culture approach enables a robust reproducible creation of a heterogeneous tumour tissue architecture with zones of proliferation, dormancy and cell death creating distinct areas of mucoepidermoid and fibroblastoid histology. Validation of such *in vitro* assays considering these aspects of modulation of antibody effects in patients are the next logical step towards improved antibody efficacy testing.

### Effects of anti-EGFR antibody treatment on skin microtissue in co-culture

**Figure 5:**
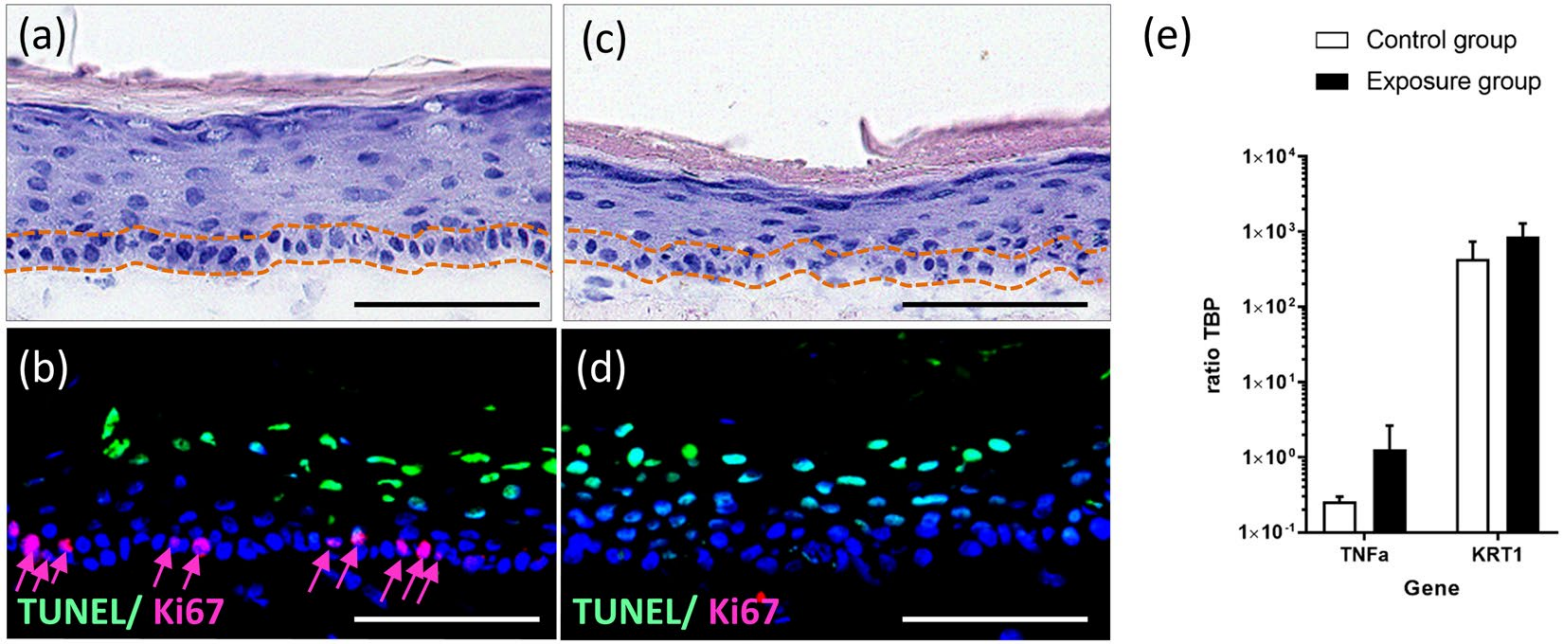
Effects of cetuximab treatment on skin microtissue at day 5 of co-culture. (**a**) H&E staining of untreated control group indicating maintained columnar basal keratinocyte layer. (**b**) Immunofluorescent TUNEL (green) and Ki67 (red, arrows) staining of untreated control group indicating proliferative keratinocytes in the basal layer. (**c**) H&E staining of antibody-exposed group showing irregular arrangement of keratinocytes in the stratum basale. (**d**) Immunofluorescent TUNEL (green) and Ki67 (red) staining of antibody-exposed group demonstrating complete lack of proliferative cells. (**a**,**c**) Dotted lines (orange) framing keratinocytes inside the stratum basale. (**b**,**d**) Nuclei stained with DAPI (blue). Scale bar: 100 µm. (**e**) Gene expression of tumour necrosis factor alpha (TNFα) and Keratin 1 (KRT1) in skin microtissue of untreated control group (white bars) and antibody exposure group (black bars), n = 3. Data shown as mean + SD.

Dermatological toxicities are the most common side effects in EGFR-targeted therapies, including a papulopustular rash, dry and itchy skin and microbial infections^5,48^. Early human cellular assays for these outcomes are lacking. Using the HMOC co-culture model, several key side effects on the cetuximab-exposed skin microtissues could be detected at a very early stage. The haematoxylin and eosin staining revealed severe damage to the arrangement of the epidermal stratum basale, seen evidently by the lacking columnar keratinocytes in the lowest layer of the epidermis (Fig. 5c, area between the orange dotted lines), accompanied by a complete loss of Ki67 positive stained keratinocytes (Fig. 5d). By comparison, morphological evaluation of untreated skin microtissues showed intact epidermal structures, including the single layer of columnar keratinocytes in the basal layer (Fig. 5a, area between the orange dotted lines) and Ki67 positive stained keratinocytes (Fig. 5b, red arrows). The epidermal structure of the untreated control was comparable to the static cultured skin model shown in Fig. 2a–c. Collagen IV expression was detected in skin microtissues of the control group and the antibody exposure group (data not shown), showing no disruptive effect on the basement membrane due to chip culture or cetuximab treatment. On the gene expression level, we noticed tendencies (not significant) of the antibody exposure group towards higher expression of TNFα and KRT1 (Fig. 5e). Epidermal-derived TNFα is a key mediator of cutaneous inflammation. Beside the regulation of immune and inflammatory responses, it also influences tissue remodelling, cell motility, cell cycle and apoptosis^49^. Therefore, increased TNFα expression could have contributed to the destroyed epidermal structures seen in cetuximab-exposed skin microtissues. KRT1 is a terminal differentiation marker indicating an altered differentiation potential. These data fit well with effects reported in literature after intensive retrospective investigations on patients treated with anti-EGFR antibodies. These antibodies are known to affect the EGFR-mediated signalling pathways by inducing growth arrest and apoptosis, decreasing cell migration, increasing cell attachment and differentiation, and stimulating inflammation^50^. This leads to impaired stratum corneum, which is the last step of a progressive disruption of a physiological epidermal turnover. The latter starts with the generation of increasingly differentiated layers of keratinocytes from basal layer stem cells. This *in vitro* safety assay senses the initial keratinocyte stem cell toxicity that occurs in patients a long time before clinical manifestations. Therefore, a prospective validation of such a sensitive microphysiological *in vitro* assay should be aspired to routinely investigate antibody-induced adverse effects while obviating animal testing.

### Cytokine release of anti-EGFR antibody treatment in the skin–tumour co-culture

**Figure 6:**
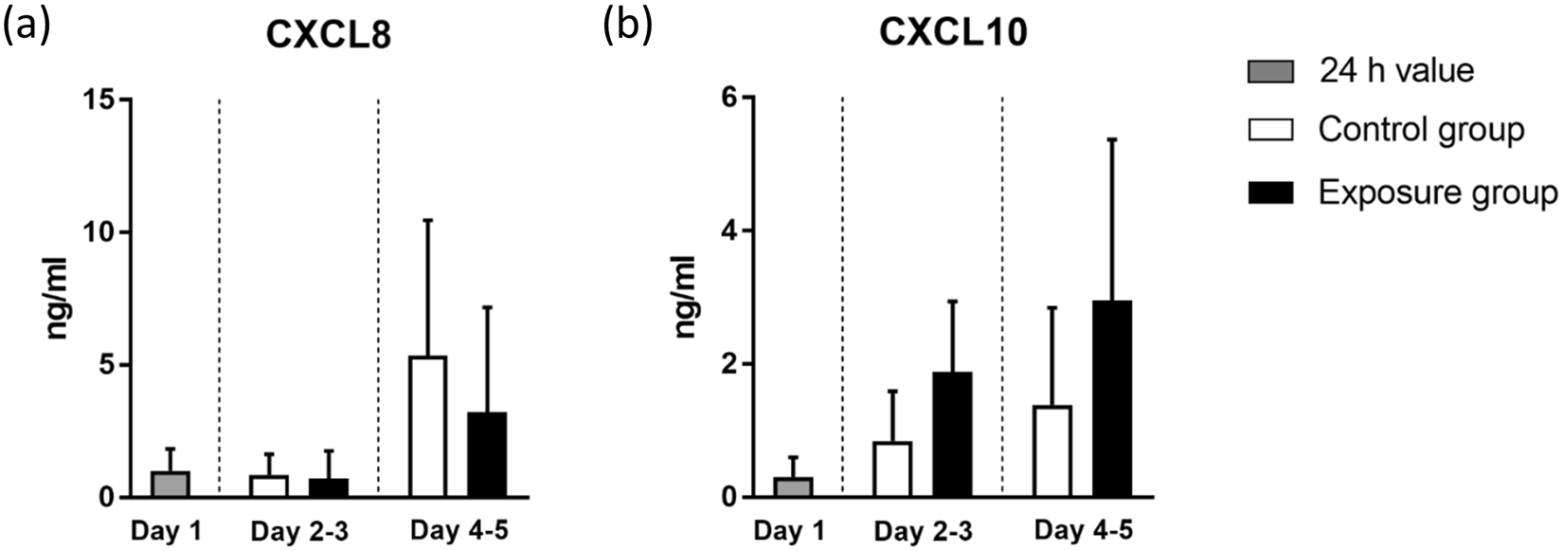
Cytokine release of chip co-cultures in cumulated periods. Values at day 1 (grey bars) indicate cytokine release within the first 24 h of co-cultures. Afterwards, mean values of respective time periods of the control group (white bars) and exposed group (black bars) are shown separately. (**a**) CXCL8. (**b**) CXCL10. n = 7–8. Data shown as mean + SD.

A set of pro-inflammatory cytokines was measured to investigate the cytokine release of anti-EGFR antibody treatment in our skin and tumour co-culture. Human full-thickness skin equivalents generally show a steady long-term pro-inflammatory cytokine excretion, which is assumed to be a continuous repair reaction due to the lack of an endothelial vasculature^51^. This effect was also observed in our study. Here we focused on the release of CXCL8 and CXCL10, two of the major pro-inflammatory cytokines associated with adverse skin effects of anti-EGFR antibody treatment^50,52^. The exposure of cetuximab reduces the release of CXCL8 in patients. Furthermore, low serum CXCL8 levels are associated with a more severe skin toxicity and a prolonged lifetime^52^. CXCL10 (also known as IFNγ-inducible protein 10) was instead shown to be increased due to anti-EGFR treatment^53,54^. In this study, CXCL8 remained at a low level in the control group and the antibody exposure group in a first phase (day 2 and 3). It then increased in both groups (day 4 and 5), with a lower release (non-significant) in the antibody exposure group (Fig. 6a). It might well be that the continuous high levels of CXCL8 contribute to the H292 tumour cell proliferation observed in the respective areas, as has been described for NSCLCs^55^. By comparison, CXCL10 increased in the control group and the antibody exposure group in all phases (day 2 to 5) with elevated levels in the antibody exposure group (Fig. 6b), correlating to the *in vivo* findings. This analysis is an essential component of our HMOC assay. Implementing those data will contribute to a complete description of safety and efficacy aspects in such MPS in the future.

Here, we introduced a microfluidic co-culture system simultaneously emulating the microenvironment of distant metastatic tumour foci in patients and human healthy skin behaviour over five days. Exposure of artificially generated 3D tumour spheroids to mild convective medium flow in one compartment enables one to sustain important naturally occurring tumour features, such as heterogeneous metastatic tissue architecture with zones of proliferation, dormancy, cell death and, eventually, high intra-tumoural pressure. The culture of a full-thickness skin equivalent at the air–liquid interface in a second interconnected compartment supports the integration of organotypic healthy organ equivalents. The modular ability of the HMOC to culture standard 24-well sized culture inserts, which introduces new possibilities for further organ combinations, is of particular interest. The commercially available MOC platform, modified for the assay presented, has already proved the maintenance of various human organotypic organ equivalents, such as intestine^56^, liver^12^, kidney^57^, neuronal tissue^58^, bone-marrow^59^ and pancreatic islets^60^, in the past. The combination of one of these human organ equivalents with any relevant tumour model, beyond the NSCLC model used here, will greatly expand the potential use of the established “safficacy” assay format into any oncological indication. Integration of vasculature^13,61^ would further improve the co-culture due to organ and tumour conditioning. The robust and reproducible combination, established here for the first time, enabled cancer-specific evaluation of efficacy and organ-specific evaluation of safety. Industrial validation of the established “safficacy” assay for the evaluation of novel anti-EGFR antibodies and their format variations, such as protobodies^38^ or low molecular weight EGFR-inhibitors, is the next logical step. Given a physiology-based pharmacokinetic compliance of upcoming MOC generations, quantitative *in vitro* to *in vivo* extrapolation might become another added value of the platform.

## Methods

### Chip design, fabrication and fluid flow modelling

The commercially available microphysiological MOC platform^12,60,62^ has been modified by enlarging one of the two 96-well culture compartments to the size of a 24-well culture compartment, thereby allowing the integration of standard 24-well-based cell culture inserts. This new type of MOC is further named the HMOC. In this study, the HMOC was used to co-culture a 24-well Millicell® standing insert-based full-thickness skin microtissue with 3D tumour microtissue spheroids (Fig. 1a). CAD software has been used for the design. The fabrication of this device was performed according to Wagner *et al.*^12^. Additionally, a pretreatment of the tumour culture compartment with Sigmacote® was implemented. A commercially produced batch of HMOCs was used for the experiments after quality control release. Pulsatile fluid flow in the tissue-connecting circuit is driven by an on-chip micropump, modified after Wu and colleagues^63^. An external control unit ensures pumping frequency, pressure and vacuum to maintain a pulsatile flow in each circuit of the HMOC. Each control unit can operate four HMOCs simultaneously. COMSOL Multiphysics R 5.2a has been applied to calculate flow rates and relevant wall shear stresses of the HMOC in different operational modes. The flow in the simulation is a steady-state Stokes flow (Re < 0.02) with a laminar inflow condition corresponding to a given flow rate and a zero-pressure outlet condition. The medium was modelled using the properties of water at 37 °C. The position and size of the mounted tumour spheroids were approximated from microscopic images to set final microfluidic flow conditions guaranteeing low shear stress for the tumour microtissues. Finally, antibody residence times in tumour microtissue areas were calculated by integrating 1/v (v: magnitude of velocity) over streamlines in a contact zone within 50 µm around the tumour microtissues.

### Characterization of fluid dynamics

We applied red blood cell microparticle image velocimetry (µPIV)^13,64^ at frequencies of 0.1 to 0.9 Hz, pressures of 200 to 800 mbar and a constant vacuum of -500 mbar. The red blood cells were prepared by centrifugation of human blood at 3,000 g for 10 min, and subsequent resuspension in phosphate-buffered saline (PBS) at a haematocrit of 2.5%. The average flow rate was determined with a high-speed CMOS camera (Baumer Optronic HXC40, Radeberg, Germany) coupled to an inverted microscope (Zeiss Axiovert, Jena, Germany). Magnification was set to 2.5×, which resulted in a resolution of 0.23 px/µm with an acquisition rate of 1,470 fps. The displacement of the red blood cells in recordings of 13.6 s was computed by the open source toolbox “PIVlab”^65^. Only velocity vectors in the central portion of the channels were considered. Details of the underlying equations have been discussed elsewhere^13^. For the rheological analyses we utilised three chips with a setup identical to the biological experiments and assessed the flow at three imaging points A, B and C (Fig. 1a). All measurements were conducted in triplicates.

### Cell sources and maintenance

Reconstructed human full-thickness skin microtissues were obtained from the Fraunhofer Institute IGB (Würzburg, Germany). Human foreskin biopsies for isolation of primary human keratinocytes and dermal fibroblasts were obtained, with informed consent from legal representatives and ethics approval (AZ-182/10, Ethic Committee of the medical faculty of the University Würzburg, Germany), after routine circumcisions in compliance with the relevant guidelines and regulations. Skin microtissues were shipped on day nine of the air–liquid culture. Upon arrival, skin tissues were transferred into 1 ml of E3 medium composed of EpiLife™ medium (MEPI500CA, Thermo Fisher Scientific Inc., Waltham, USA) supplemented with human keratinocyte growth supplement, including 0.2 ng/ml human epidermal growth factor (S0015, Thermo Fisher Scientific Inc.), 1.4 mM CaCl_2_, 50 µg/ml ascorbic acid (Sigma–Aldrich, St. Louis, MO, USA) and 10 ng/ml keratinocyte growth factor (Preprotech, Hamburg, Germany). After a resting phase of 24 h at 37 °C and 5% CO_2,_ the 24-well Millicell® standing inserts containing the skin microtissues were transferred as a whole into the 24-well culture compartment of the HMOC. Additional skin tissues were maintained unchanged under static culture conditions for morphological evaluation.

The human lung tumour cell line NCI-H292 (H292, American Type Culture Collection ATCC® CRL1848™, Manassas, VA, USA), used in standard two-dimensional (2D) monolayer efficacy testing, were grown in RPMI1640 medium supplemented with 10% foetal calf serum, 50 µg/ml gentamycin sulphate and 0.25 µg/ml Amphotericin B (Corning, Lowell, MA, USA). Standard 384-well ultra-low attachment spheroid microplates (Corning) were used for the formation of H292 lung tumour spheroids. Briefly, 50 µl of medium containing 10,000 H292 cells was pipetted into each well of the microplates. The microplates were centrifuged at 300 g for 1 min at room temperature and then incubated on a 3D rotator (PS-M3D, Grant, Shepreth, UK) at 37 °C and 5% CO_2_. Compact tumour spheroids formed within three days. Fifteen spheroid microtissues were then transferred to the tumour culture compartment of the HMOC. Additional tumour microtissues were embedded in Tissue-Tek® O.C.T.™ Compound (Sakura, Alphen aan den Rijn, Netherlands) and subsequently snap-frozen for morphological evaluation of preformed tumour spheroids.

### Cetuximab proliferation assay

A static monolayer proliferation assay was performed for the evaluation of cetuximab-dependent cytotoxicity of H292 cells. Thus, 5000 H292 lung tumour cells were seeded per well in a 96-well plate using 100 µl of supplemented RPMI1640 medium. The next day, a concentration series of cetuximab was applied originating from a stock solution of 15.5 mg/ml (Bayer AG, Wuppertal, Germany). Cells were incubated for additional 96 h followed by a standard MTT (3-(4,5-dimethylthiazol-2-yl)-2,5-diphenyltetrazolium bromide) viability assay. Briefly, 20 µl of MTT reagent (stock solution 5 mg/ml, dissolved in PBS) was added to each well and incubated for 2 h at 37 °C (5% CO_2_). Afterwards, the supernatant was aspirated and 130 µl of lysis buffer, consisting of Isopropanol (VWR Int., Fontenay-sous-Bois, France) and 3% sodium dodecyl sulphate (Carl-Roth, Karlsruhe, Germany), was applied and incubated for an additional 15 min. Absorbance was measured using a microplate reader (FLUOstar Omega, BMG Labtech, Ortenberg, Germany) at 570 nm. Mean absorbance of the control group was set to 100% of viability for subsequent calculations.

### HMOC-based co-culture assay

One full-thickness skin microtissue and 15 tumour microtissues were loaded consecutively into the respective culture compartments of each HMOC circuit (Fig. 1a) for a five-day co-culture (Fig. 2b). Each was conducted in 1 ml E3 medium, supplemented with additional 0.5 g/l of D(+)-glucose (Carl-Roth), resulting in a final concentration of 1.5 g/l glucose. A complete medium exchange in both compartments was performed after 24 h of co-culture in two groups: the control group and the antibody exposure group (n = 4). Subsequently, half of the medium was exchanged daily. The latter half medium exchange was applied with a concentration of 3 g/l glucose. The E3 medium contained 10 µg/ml anti-EGFR antibody for the antibody exposure group. Co-cultures were completed after five days and the microtissues were collected for further analysis.

### Daily tissue culture analysis

Pictures of lung spheroids cultured inside the tumour culture compartment of the HMOC were taken daily using the BZ-9000 Microscope with the BZ-II-Viewer software and analysed using the BZ-II-Analyser software (Keyence, Neu Isenburg, Germany) for the evaluation of tumour microtissue remodelling.

Furthermore, tissue viability was monitored daily by the measurement of lactate dehydrogenase (LDH) released in the supernatant using the Cytotoxicity Detection Kit^PLUS^ (Roche Diagnostics, Mannheim, Germany). Measurement was performed according to the manufacturer’s instructions, with minor modifications: 12.5 µl of reaction mix was added to 12.5 µl of prediluted sample into 384-well non-treated microplates (Corning) and incubated for 30 min at room temperature. Photometric measurement took place using a microplate reader at 490 and 680 nm. Calculations were conducted via MARS Data Analysis Software and further analysed using GraphPad Prism® 7 software.

### Cytokine quantification in cell culture supernatants

Medium was collected from the cell cultures at the time points indicated and stored at −80 °C. Samples were thawed and centrifuged to remove any debris on the day of analysis. Chemokines were quantified in the supernatants using the V-PLEX Chemokine Panel 1 Human Kit, on the QuickPlex Meso SQ 120 (Meso Scale Diagnostics, LLC), according to the kit manuals.

### End-point tissue culture analyses

Skin and tumour microtissues from each culture condition were embedded in Tissue-Tek® O.C.T.™ Compound and subsequently snap frozen for end-point analysis. Tissue blocks were sectioned with a Leica CM1950 Cryostat (Wetzlar, Germany) at a thickness of 8 µm. Standard haematoxylin and eosin staining was used to analyse the sections histologically. A combined TUNEL (TdT-mediated dUTP-digoxigenin nick end labeling)/Ki67 immunofluorescence staining was performed to detect proliferating and apoptotic cells. Thus, representative cryostat central sections of the microtissues were stained using the Apo Direct Apoptosis Detection Kit (Thermo Fischer Scientific Inc.), according to the manufacturer’s instructions. Subsequently, slides were blocked with 10% (v/v) goat serum in PBS for 20 min and primary mouse anti-human Ki67 antibody (Thermo Fischer Scientific Inc.) was applied overnight, washed with PBS and then developed by goat anti-mouse CF594 (Biotium, Fremont, CA, USA) for 45 min. DAPI (Roche) was added for nuclei staining. Further single immunofluorescence staining on skin microtissue was performed using mouse anti-human Collagen IV antibody (Sigma–Aldrich) combined with goat anti-mouse CF594 for visualisation of the basement membrane on skin sections. Tumour microtissue sections were single stained with rabbit anti-human Vimentin (Thermo Fisher Scientific Inc.) or mouse anti-human E-Cadherin antibody with the appropriate secondary antibody, goat anti-rabbit CF594 or goat anti-mouse CF594 (Biotium). A Keyence BZ-9000 Microscope with BZ-II-Viewer software was used for microscopic imaging. Images were merged using the BZ-II-Analyser software. DAPI and Ki67 positively stained cells were counted manually using this software to determine the proportion of proliferative cells.

Real-time qPCR endpoint analysis was performed to evaluate tumour and skin microtissue gene transcription at an mRNA level. Thus, microtissues from both culture conditions were collected for RNA isolation using the NucleoSpin RNA Kit (Macherey-Nagel, Düren, Germany). cDNA was synthesized by reverse transcription of 100 ng total RNA using the TaqMan® Reverse Transcription Kit (Thermo Fisher Scientific Inc.). Quantitative PCR was performed using the QuantStudio 5 Real-Time PCR System (Thermo Fisher Scientific Inc.) and the SensiFAST SYBR Lo-ROX Kit (Bioline, Luckenwalde, Germany), according to the manufacturer’s instructions. The real-time qPCR primers were as follows: tumour protein 53 (p53) forward 5′-TATGAGCCGCCTGAGGTTGG-3′ and reverse 5′-GGCACAAACACGCACCTCAA-3′ Bcl-2-associated X protein (BAX) forward 5′-CAACCACCCTGGTCTTGGAT-3′ and reverse 5′-CTGACGGCAACTTCAACTGG-3′, tumour necrosis factor alpha (TNFα) forward 5′-CTCGAACCCCGAGTGACAAG-3′ and reverse 5′-ATGGTGTGGGTGAGGAGCAC-3′, Keratin 1 (KRT1) forward 5′-GCTGGCAGACATGGGGATAG-3′ and reverse 5′-CATCCTTGAGGGCATTCTCG-3′, Glyceraldehyde 3-phosphate dehydrogenase 5′-TGTTGCCATCAATGACCCCTT-3′ and reverse 5′-CTCCACGACGTACTCAGCG-3′ and TATA-binding protein (TBP) forward 5′-CCTTGTGCTCACCCACCAAC-3′ reverse 5′-TCGTCTTCCTGAATCCCTTTAGAATAG-3′.

### Statistical analysis

Differences among the control group and the antibody exposure group were analysed using unpaired two-tailed Student’s t test and a p value < 0.05 was considered statistically significant.

## Data Availability

The datasets generated during and/or analysed during the current study are available from the corresponding author on reasonable request.
